# Efficacy and Safety of Nanoparticle Loteprednol Etabonate Compared to Vehicle in Post-cataract Surgery Pain and Anterior Chamber Inflammation Management: A Systematic Review and Meta-Analysis

**DOI:** 10.7759/cureus.71266

**Published:** 2024-10-11

**Authors:** Hatem M Alsolami, Ali S Alsudais, Mohammad H Nooh, Basel M Alsolami, Mohammed M Alghamdi, Naif Almufarriji, Saeed A Alghamdi

**Affiliations:** 1 College of Medicine, King Saud Bin Abdulaziz University for Health Sciences, Jeddah, SAU; 2 Medical Research, King Abdullah International Medical Research Centre, Jeddah, SAU; 3 Department of Ophthalmology, Makkah Healthcare Cluster, Ministry of Health, Makkah, SAU; 4 Ophthalmology, King Abdulaziz Medical City, Ministry of National Guard Health Affairs, Jeddah, SAU; 5 Ophthalmology, Department of Surgery, Division of Ophthalmology, Ministry of National Guard Health Affairs, Jeddah, SAU

**Keywords:** aging, anterior chamber, cataract surgery, nanoparticle-based loteprednol etabonate, pain

## Abstract

Cataracts are a leading cause of blindness worldwide, with phacoemulsification being the most widely used surgical method for cataract extraction due to its proven effectiveness. Managing postoperative inflammation following ocular surgery is crucial, and topical ocular corticosteroids have become an essential part of this therapeutic approach. This review aims to evaluate the efficacy and safety of nanoparticle-sized loteprednol etabonate (LE) formulations in comparison to a placebo, utilizing a systematic review and meta-analysis.

We conducted a comprehensive search across Cochrane Central Register of Controlled Trials (CENTRAL), Medical Literature Analysis and Retrieval System Online (MEDLINE), EBSCO, and ClinicalTrials.gov. Only randomized controlled trials (RCTs) that compared nanoparticle-sized LE formulations with placebo were included. Efficacy was measured by the resolution of anterior chamber cells (ACC) and anterior chamber flare (ACF), as well as pain relief. Safety was assessed by tracking adverse events (AEs). The risk of bias was evaluated using the Cochrane risk of bias tool. Eight RCTs involving 2,375 participants met our criteria.

The meta-analysis revealed that both 0.38% and 1.00% LE formulations significantly improved ACC resolution, with the 1.00% formulation showing a higher efficacy (relative risk (RR)=1.87, 95% confidence interval (CI): 1.58-2.2, P<0.01, I²=40%). Furthermore, the 1.00% LE formulation was more effective in relieving pain compared to placebo (RR=1.52, 95% CI: 1.39-1.66, P<0.01, I²=20%). Both LE formulations were associated with few adverse events, and the 1.00% LE formulation had a lower risk of such events compared to placebo. However, no significant differences were observed in terms of joint swelling, stiffness, or mortality.

In conclusion, our meta-analysis supports the use of nanoparticle-sized LE for managing inflammation and pain post-cataract surgery, with the 1.00% formulation proving to be more effective than both 0.38% LE and placebo. Both formulations were found to be generally safe, though additional research is needed to further evaluate long-term outcomes and safety.

## Introduction and background

Cataracts are a common ocular disorder characterized by the opacification of the crystalline lens, which poses a significant risk for visual impairment and eventual blindness [1]. The lens is derived from ectodermal tissue and comprises epithelial cells that continuously proliferate and differentiate into lens fibers throughout an individual's lifespan, resulting in the progressive thickening and compaction of the lens structure with advancing age [2]. While aging is the primary etiological factor contributing to cataract development, other factors can also play a role, such as disease, trauma, medication, and genetic predisposition [1,3].

Age-related changes in the lens contribute to cataract pathogenesis through multiple mechanisms. Firstly, the accumulation of yellow-brown pigments within the lens due to aging hampers light transmission. Secondly, structural modifications occur in the lens fibers, disrupting their regular architecture and arrangement, which is vital for maintaining optimal optical clarity [3].

Cataract represents a significant cause of blindness globally, affecting approximately 65.2 million individuals and accounting for 51% of all blindness cases. Their burden affects developed and developing nations, but higher prevalences have been reported in underdeveloped or isolated regions [4]. Bilateral blindness affects nearly 18 million individuals, whereas cataracts contribute to moderate to severe vision loss in 52.6 million [4]. The prevalence of cataracts among individuals aged ≥40 years ranges from 11.8% to 18.8% [4].

In developed countries, phacoemulsification is the most commonly performed surgical technique for cataract removal due to its established efficacy [1,3]. More than 26 million cataract surgeries are conducted annually worldwide, increasing at a compound annual growth rate of 3.1%. This growth can be attributed to demographic shifts and the expansion of medical services. The evolution of cataract surgery has progressed from earlier methods, such as intracapsular cataract extraction and extracapsular cataract extraction, to the current gold standard phacoemulsification procedure, which has been widely adopted in developed nations [4].

Mechanical stress during eye surgery can trigger an inflammatory response characterized by membrane breakdown and tissue damage. If this inflammatory reaction is not effectively managed, it can elevate the risk of postoperative discomfort, edema, erythema, anterior chamber cells (ACCs) and flare (ACF), secondary glaucoma, posterior synechia, and potentially cystoid macular edema [5-7]. Despite advancements in intraocular lens implants and surgical techniques, some degree of postoperative inflammation still occurs after cataract surgery. Therefore, effectively controlling and preventing pain and inflammation resulting from cataract surgery is crucial to enhance the overall patient experience [6-9].

Topical ocular corticosteroids have emerged as a critical therapeutic modality for managing postoperative inflammation after ocular surgery. These agents effectively alleviate symptoms such as photophobia, edema, discomfort, and soreness [7]. Loteprednol etabonate (LE), a carbon-20 ester corticosteroid, has been formulated in various topical ocular preparations for postoperative use, including solutions, ointments, and gels. LE exhibits potent anti-inflammatory properties while carrying a low risk of adverse side effects, such as elevated intraocular pressure [7-10]. An intriguing characteristic of LE is its unique C-20 ester group, contrasting with the C-20 ketone group present in other glucocorticoids. This structural modification imparts specific properties to LE, enabling it to undergo rapid de-esterification by widely distributed esterases. Consequently, LE undergoes enzymatic hydrolysis upon binding to the glucocorticoid receptor, forming two inactive metabolites [10].

Recent studies have found that reducing the particle size in the LE gel to the nanometer range, resulting in a 0.38% and 1.00% concentration confers significant advantages in ocular tissue penetration and resolution of inflammation and pain. Despite having a lower drug concentration, this refined formulation exhibits superior ocular penetration and efficacy than a micronized 0.50% LE gel. These observations substantiate the potential of the nanosized LE gel formulation to reduce medication frequency, enhance patient compliance, and ultimately optimize clinical outcomes [7,10].

The efficacy and safety of 0.38% and 1.00% LE in treating postoperative inflammation patients after cataract surgery have been extensively investigated in randomized clinical trials [11-15]. Therefore, we conducted a systematic review and meta-analysis to assess the comparative effectiveness and safety of 0.38% and 1.00% LE in managing pain and inflammation after cataract surgery.

## Review

Protocol

This study conducted a comprehensive and rigorous systematic review based on a predetermined protocol registered with PROSPERO (CRD42023425440). It adhered to the Preferred Reporting Items for Systematic Reviews and Meta-Analysis (PRISMA) checklist to ensure transparent and accurate reporting of its methods and findings. 

Study selection and data extraction

Two reviewers (AS and MG) independently screened the titles and abstracts, comprehensively evaluated the full text, and extracted data from randomized controlled trials (RCTs), ensuring a rigorous and unbiased analysis. Any inconsistencies or conflicts during the screening and evaluation were resolved through discussion with a third reviewer until a consensus was reached, ensuring definitive decisions.

Risk of bias assessment

Two reviewers (MN and HA) independently evaluated the risk of bias in the included RCTs using the revised Cochrane Risk of Bias tool [16]. The risk of bias was deemed high in two RCTs and low in two RCTs, while there were some concerns in the remaining RCT. Reviewer conflicts were resolved through discussion to achieve consensus. The quality of evidence for each outcome was evaluated using the Grading of Recommendations Assessment, Development, and Evaluation (GRADE) criteria [17]. The GRADE instrument, which is endorsed by the Cochrane Collaboration, was used to assess the quality of evidence, and determine the strength of recommendations in the studies included in the meta-analysis [18]. This comprehensive evaluation considered important factors such as research design, consistency, indirectness, heterogeneity, imprecision, publication bias, and other relevant characteristics described in the papers reviewed. The quality of evidence was then categorized into different levels - high, moderate, low, or very low - based on the overall assessment using the GRADE framework [17,18].

Meta-analysis

Meta-analysis was performed using RevMan (version 5.3), a software tool developed by the Cochrane Collaboration, strictly following established academic protocols. The meta-analysis used a random effects model, which effectively accounts for the heterogeneity inherent among the included RCTs. Statistical significance was determined at a confidence level of 95% and a significance threshold of P < 0.05. Risk ratios (RRs) were used as the effect measure for dichotomous outcomes such as ACCs, ACF, Grade 0 Pain, adverse events (AEs), and serious AEs (SAEs), and a pooling method based on inverse variance weighting was implemented. Subgroup analyses were further performed to explore potential differences in the postoperative timing of patient assessments in the intervention and placebo groups.

Results

**Figure 1:**
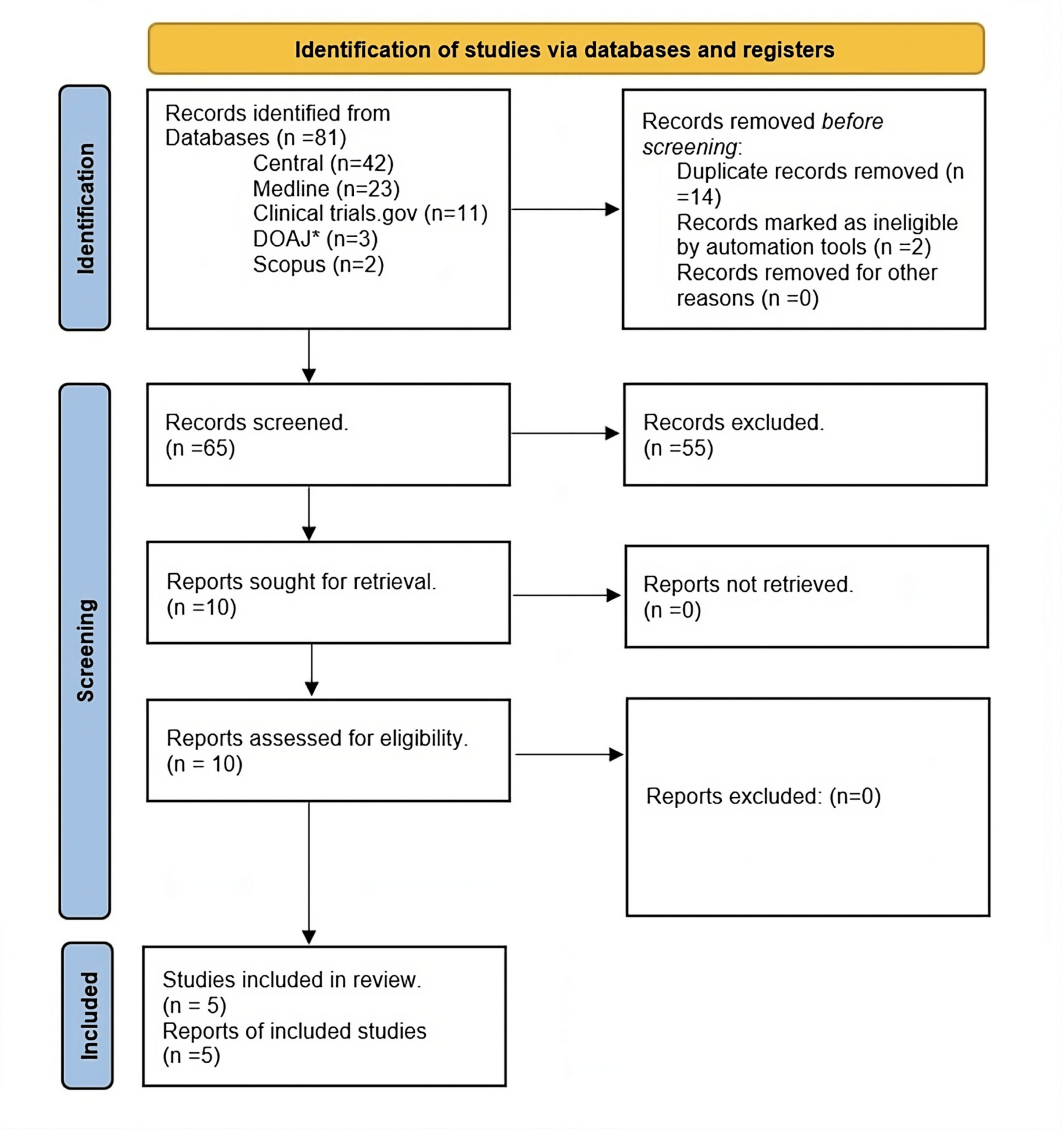
PRISMA flowchart A flow diagram outlining the process of study selection for this meta-analysis was created following the guidelines set by the Preferred Reporting Items for Systematic Reviews and Meta-Analyses (PRISMA)

The systematic search identified 81 articles that were initially retrieved. After removing 14 duplicates, 65 unique articles remained. Among them, 55 were deemed ineligible and excluded based on the predefined criteria. Ultimately, five articles corresponding to five distinct RCTs met the eligibility requirements and were included in the analysis. The selection process is visually presented in Figure 1.

Trial characteristics

The RCTs involved 2340 participants, with a mean age of 67.4 to 69.8 years. Among them, 1078 were male (46.1%), and 1254 were identified as white (53.6%), with the remaining participants representing diverse racial backgrounds, including American Indian, Asian, Native Hawaiian or other Pacific Islander, Black or African American, and mixed race. However, the race of some participants was unknown or unreported. The baseline characteristics of the participants included in the study are presented in Table 1.

**Table 1:** Trial characteristics This table shows the demographics and baseline characteristics of participants in all the included trials, including the number of participants, gender, race, mean age, drug delivery type, and dose.

Trial Registry	Delivery type	Dose (%)	Number of participants (started)		Number of participants (completed)		Gender		Race							Mean age
			Loteprednol Etabonate Gel	Vehicle	Loteprednol Etabonate Gel	Vehicle	male	female	American Indian or Alaska Native	Asian	Native Hawaiian or Other Pacific Islander	Black or African American	White	More than one race	Unknown or Not Reported	
NCT02208297 [15]	Nanoparticle Gel	0.38	163	163	141	107	136	190	0	0	0	0	0	0	326	67.4 (9.19)
NCT01996839 [11]	Nanoparticle Gel	0.38	342	172	272	80	271	283	0	0	0	0	0	0	514	69.8 (8.36)
NCT02786901 [12]	Nanoparticle Gel	0.38	401	199	235	304	237	363	2	29	1	60	490	2	16	68.2 (9.11)
NCT02163824 [13]	Nano particle suspension	1	254	126	251	124	154	226	0	61	5	30	274	2	8	68.7 (34 to 89)
NCT02793817 [14]	Nano particle suspension	1	261	259	258	254	232	288	2	36	4	51	413	2	12	68.4 (38 to 90)

Risk of bias assessment

**Figure 2:**
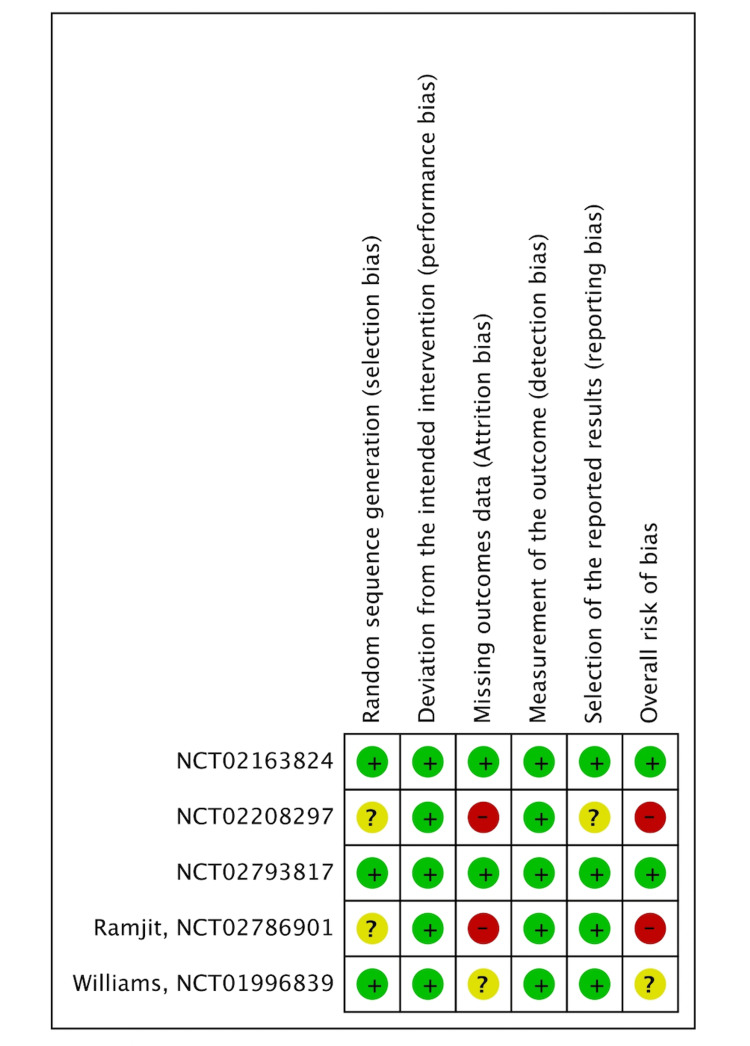
Risk of bias Summary Study or subgroup: NCT02163824 [13]; NCT02208297 [15]; NCT02793817 [14]; NCT02786901 [12]; NCT01996839 [11]

Two of the five RCTs had a low risk of bias, one had some concerns, and two had a high risk of bias. Figure 2 shows the assessment of the risk of bias in all the included RCTs. 

Efficacy

ACC Grade

**Figure 3:**
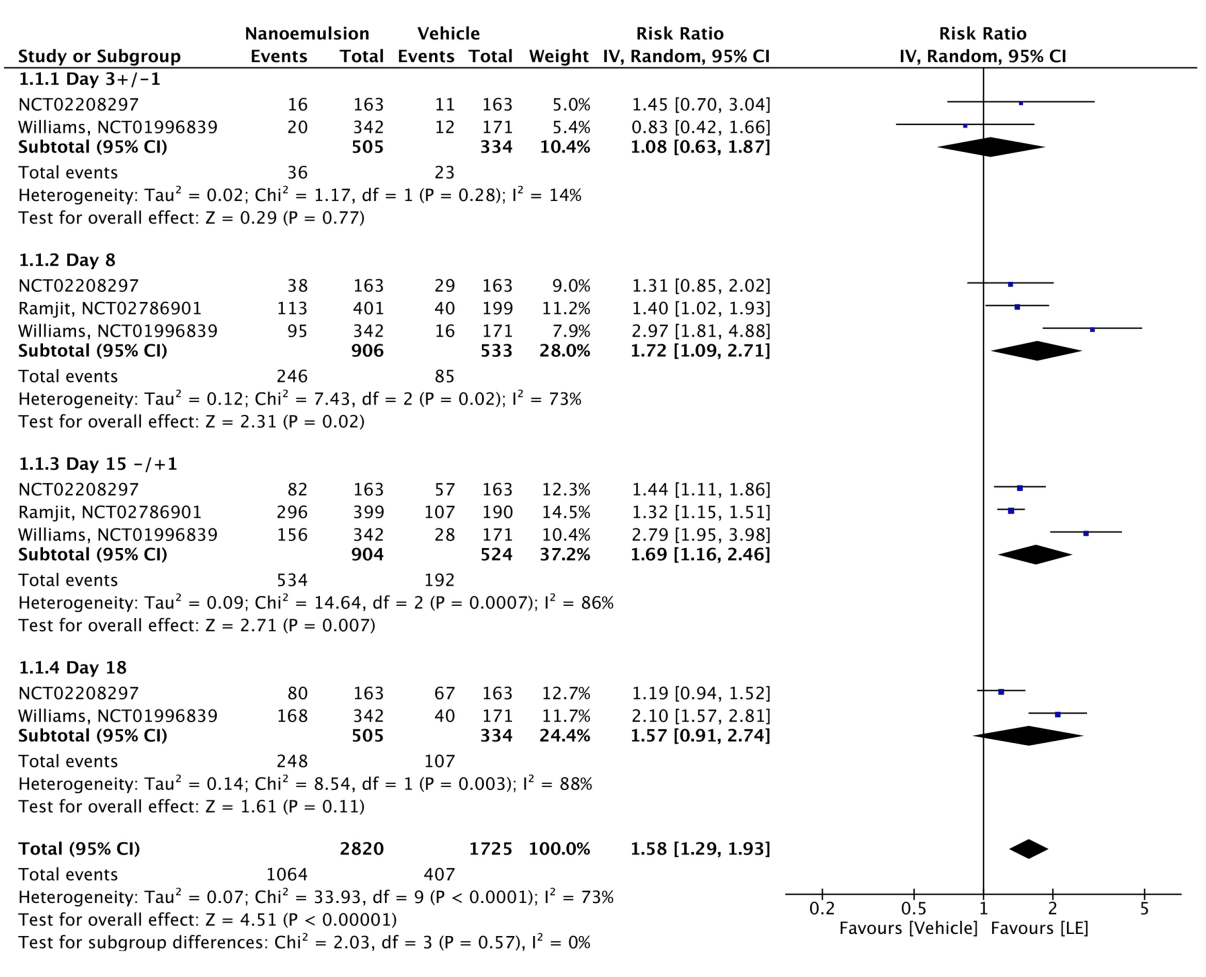
Forest plot of ACC grade of 0.38% LE ACC: anterior cell champer; LE: loteprednol etabonate, CI: confidence interval; IV: inverse variance; SD: standard deviation Study or subgroup: NCT02208297 [15]; NCT02786901 [12]; NCT01996839 [11]

The proportion of patients with complete ACC resolution has been measured. The five included RCTs compared complete ACC resolution rates between participants treated with the vehicle and those treated with 0.38% or 1.00% LE. All three RCTs that used the 0.38% LE gel included ACC clearance as an efficacy measure. The meta-analysis showed that 0.38% LE gel significantly improved anterior chamber inflammation after cataract surgery but with considerable heterogeneity (RR = 1.58, 95% confidence interval [CI] = 1.29-1.93, P < 0.00001; I² = 73%). Subgroup analyses of ACCs showed RRs of 1.08 on postoperative day 3 ± 1 (95% CI = 0.63-1.87, P = 0.770; I² = 14%), 1.72 on day 8 ± 1 (95% CI = 1.09-2.71, P = 0.020; I² = 73%), 1.69 on day 15 ± 1 (95% CI = 1.16-2.46, P = 0.007; I² = 86%), and 1.57 on day 18 ± 1 (95% CI = 0.91-2.74, P = 0.110, I² = 88%). However, these results showed significant and substantial heterogeneity (Figure 3). The evidence was assessed with high certainty, as shown in Table 2.

**Table 2:** GRADE criteria assessment Grading of Recommendations Assessment, Development, and Evaluation (GRADE) evidence profile a: High risk of bias. b: High heterogeneity; ACC: anterior cell champer; ACF: anterior cell flare; LE: loteprednol etabonate

Certainty assessment							Certainty
Outcomes	Study Design	Risk of bias	Inconsistency	Indirectness	Imprecision	Other considerations	
ACC grade of 0.38% LE	Randomized Trials	Serious^a ^	Serious ^b^	Not Serious	Not Serious	Very Strong Association	High
ACC grade of 1.00% LE	Randomized Trials	Not Serious	Not Serious	Not Serious	Not Serious	Very Strong Association	High
ACF	Randomized Trials	Serious^a ^	Serious ^b^	Not Serious	Not Serious	Very Strong Association	High
Combined ACC and ACF	Randomized Trials	Serious^a ^	Serious ^b^	Not Serious	Not Serious	Strong Association	Moderate
Grade 0 (no) pain of 0.38% LE	Randomized Trials	Serious^a ^	Not Serious	Not Serious	Not Serious	Very Strong Association	High
Grade 0 (no) pain of 1.00% LE	Randomized Trials	Not Serious	Not Serious	Not Serious	Not Serious	Very Strong Association	High
Safety profile of 0.38% LE	Randomized Trials	Serious^a ^	Not Serious	Not Serious	Not Serious		Moderate
Safety profile of 1.00% LE	Randomized Trials	Not Serious	Not Serious	Not Serious	Not Serious		High

**Figure 4:**
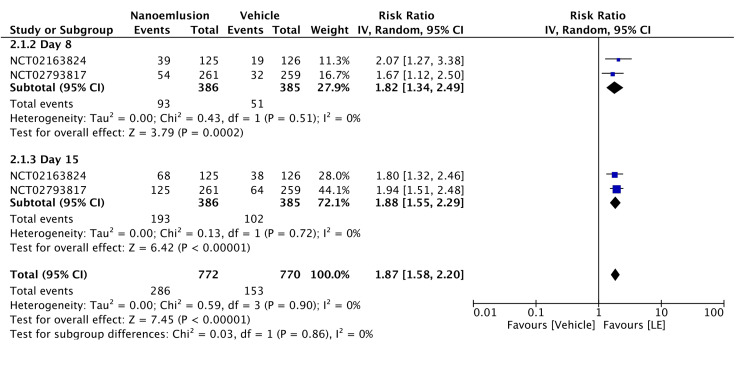
Forest plot of ACC grade of 1.00% LE ACC: anterior cell champer; LE: loteprednol etabonate, CI: confidence interval; IV: inverse variance; SD: standard deviation Study or subgroup: NCT02163824 [13]; NCT02793817 [14]

The topical application of 1.00% LE gel significantly reduced anterior chamber inflammation after cataract surgery (RR = 1.87, 95% CI = 1.58-2.2, P < 0.00001; I² = 0%). Subgroup analyses showed RRs of 1.82 on day 8 ± 1 (95% CI = 1.34-2.49, P < 0.00002; I² = 0%) and 1.88 on day 15 ± 1 (95% CI = 1.55-2.29, P < 0.00001; I² = 0%) (Figure 4). The evidence was assessed with high certainty, as shown in Table 2. 

ACF

**Figure 5:**
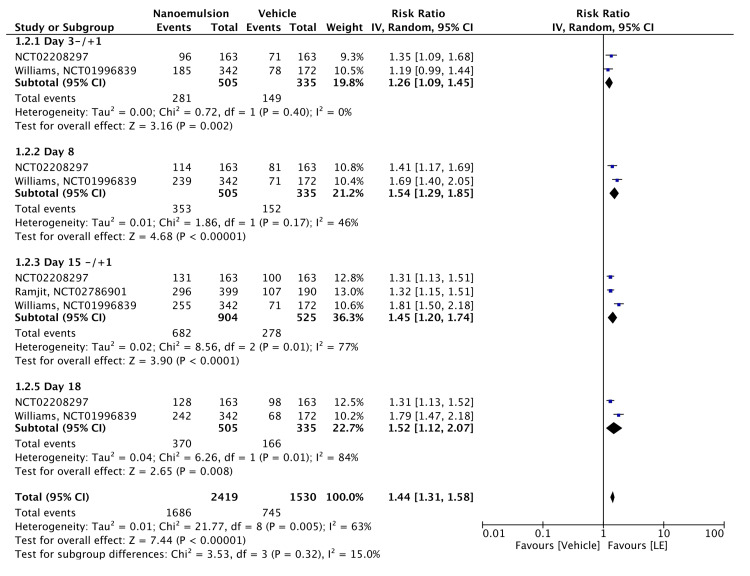
Forest plot of ACF ACF: anterior cell flare; LE: loteprednol etabonate; CI: confidence interval; IV: inverse variance; SD: standard deviation Study or subgroup: NCT02208297 [15]; NCT02786901 [12]; NCT01996839 [11]

Significantly more patients receiving the 0.38% LE gel achieved complete ACF resolution than those receiving the vehicle in the postoperative period (RR = 1.44, 95% CI = 1.31-1.58, P < 0.00001; I² = 63%). Subgroup analyses showed RRs of 1.26 for day 3 ± 1 (95% CI = 1.09-1.45, P = 0.002, I² = 0%), 1.54 for day 8 ± 1 (95% CI = 1.29-1.85, P < 0.00001; I² = 46%), 1.45 for day 15 ± 1 (95% CI = 1.20-1.74, P < 0.00001; I² = 77%), and 1.52 for day 18 ± 1 (95% CI = 1.12-2.07, P = 0.008; I² = 84%). All RRs were statistically significant, although heterogeneity was notably higher on days 15 and 18 (Figure 5). The evidence was assessed with high certainty, as shown in Table 2.

Combined ACC and ACF

**Figure 6:**
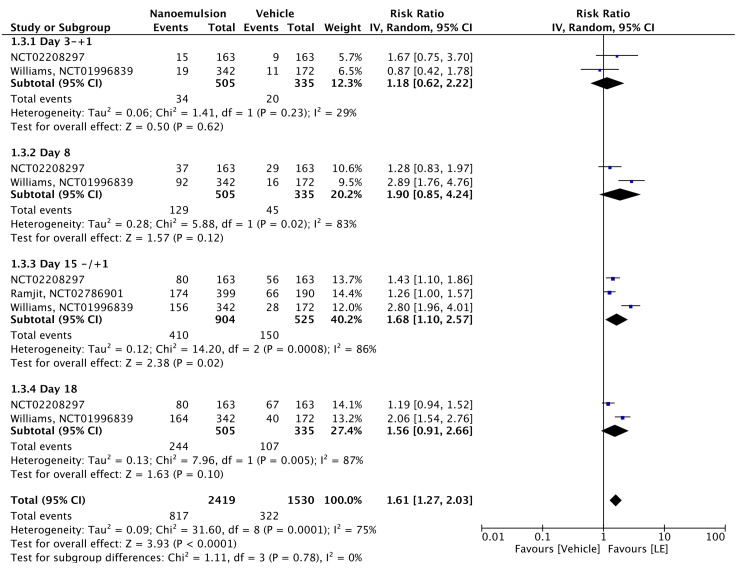
Forest plot of Combined ACC and ACF ACC anterior cell champer; ACF: anterior cell flare; LE: loteprednol etabonate; CI: confidence interval; IV: inverse variance; SD: standard deviation Study or subgroup: NCT02208297 [15]; NCT02786901 [12]; NCT01996839 [11]

Three of the included RCTs assessed combined ACC and ACF resolution with 0.38% LE gel. The meta-analysis revealed significant but highly heterogeneous results (RR = 1.61, 95% CI = 1.27-2.03, P < 0.00001; I² = 75%). Subgroup analyses showed a nonsignificant RR on day 3 ± 1 (RR = 1.18, 95% CI = 0.62-2.22, P = 0.620; I² = 29%) but significant RRs on days 8 ± 1 (RR = 1.90, 95% CI = 0.85-4.24, P = 0.120; I² = 83%), 15 ± 1 (RR = 1.68, 95% CI = 1.10-2.57, P = 0.020; I² = 86%), and 18 ± 1 (RR = 1.56, 95% CI = 0.91-2.66, P = 0.005; I² = 87%), albeit with varying degrees of heterogeneity (Figure 6). The evidence was assessed with high certainty, as shown in Table 2.

Grade 0 (No) Pain

**Figure 7:**
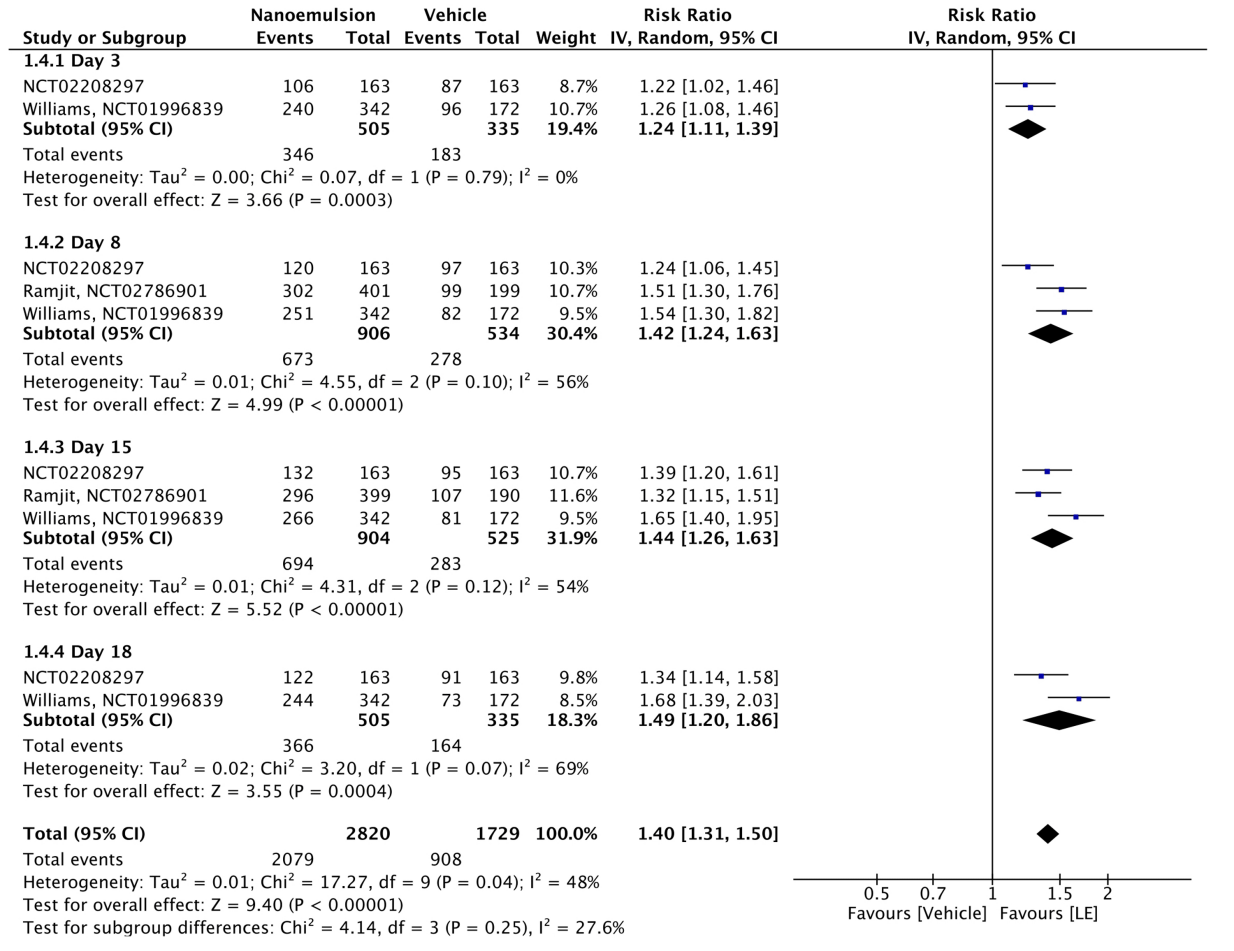
Forest plot of Grade 0 (no) pain of 0.38% LE LE: loteprednol etabonate, CI: confidence interval; IV: inverse variance; SD: standard deviation. Study or subgroup: NCT02208297 [15]; NCT02786901 [12]; NCT01996839 [11]

Ocular pain completely resolved within the postoperative period in all 2340 participants (grade = 0). The meta-analysis showed a significantly higher likelihood of pain resolution with 0.38% LE gel than with the vehicle (RR = 1.40, 95% CI = 1.31-1.50, P < 0.00001; I² = 48%). Subgroup analyses revealed significantly improved pain resolution on days 3 ± 1 (RR = 1.24, 95% CI = 1.11-1.39, P = 0.0003; I² = 0%), 8 ± 1 (RR = 1.24, 95% CI = 1.24-1.63, P < 0.00001; I² = 56%), and 15 ± 1 (RR = 1.44, 95% CI = 1.26-1.63, P < 0.00001; I² = 54%) (Figure 7). The evidence was assessed with high certainty, as shown in Table 2.

**Figure 8:**
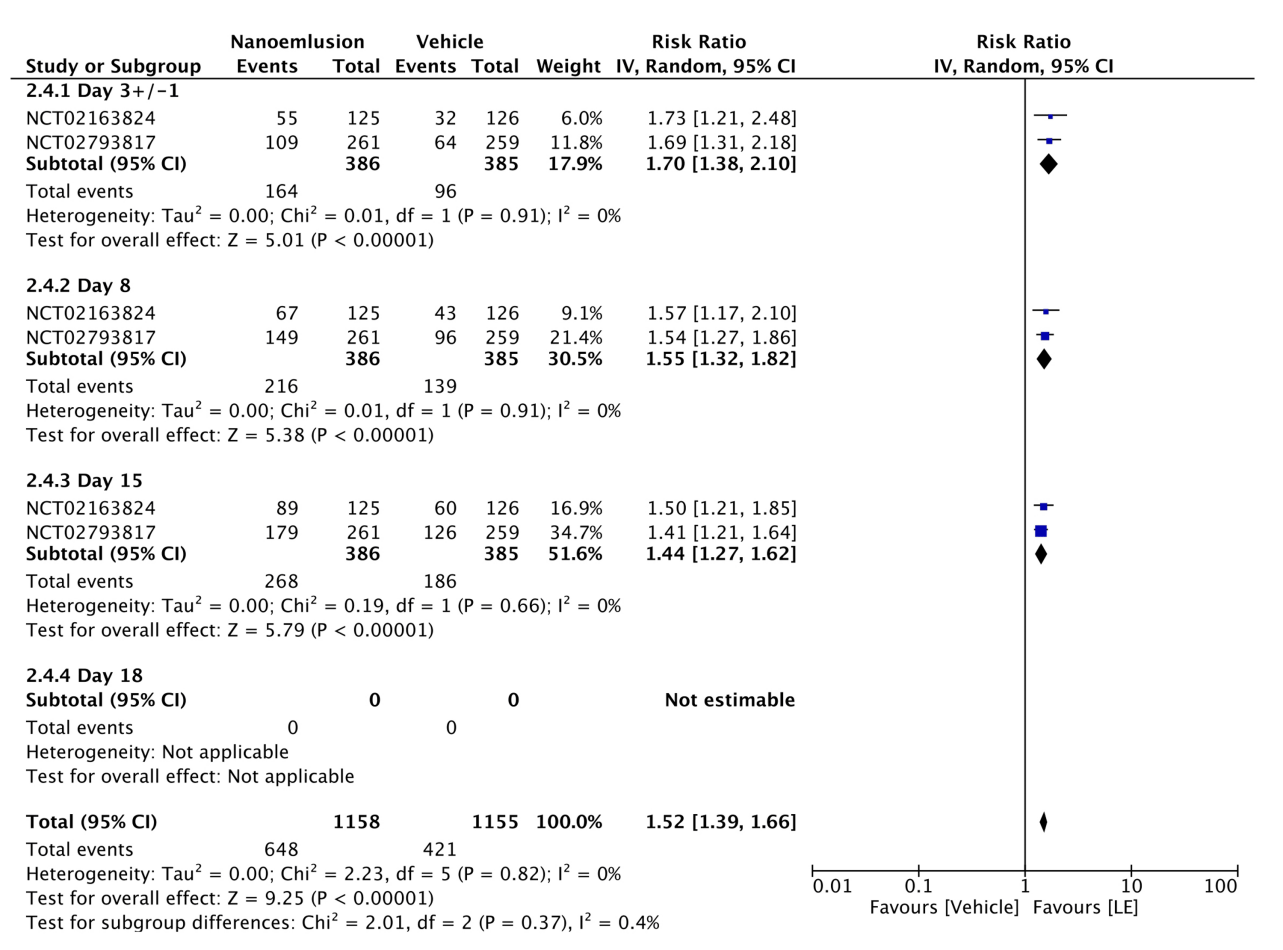
Forest plot of Grade 0 (no) pain of 1.00% LE LE: loteprednol etabonate, CI: confidence interval; IV: inverse variance; SD: standard deviation Study or subgroup: NCT02163824 [13]; NCT02793817 [14]

The topical application of 1.00% LE gel significantly improved pain resolution after cataract surgery (RR = 1.52, 95% CI = 1.39-1.66, P < 0.00001; I² = 0%). Subgroup analyses revealed significantly improved pain resolution with the 1.00% LE gel on days 3 ± 1 (RR = 1.70, 95% CI = 1.38-2.10, P < 0.00001; I² = 0%), 8 ± 1 (RR = 1.55, 95% CI = 1.32-1.82, P < 0.00001; I² = 0%), and 15 ± 1 (RR = 1.88, 95% CI = 1.55-2.29, P < 0.00001; I² = 0%) (Figure 8). The evidence was assessed with high certainty, as shown in Table 2.

Safety Profile

**Figure 9:**
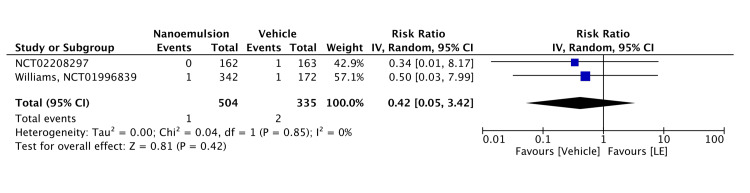
Forest plot of safety profile of 0.38% LE LE: loteprednol etabonate, CI: confidence interval; IV: inverse variance; SD: standard deviation Study or subgroup:NCT02208297 [15]; NCT01996839 [11]

**Figure 10:**
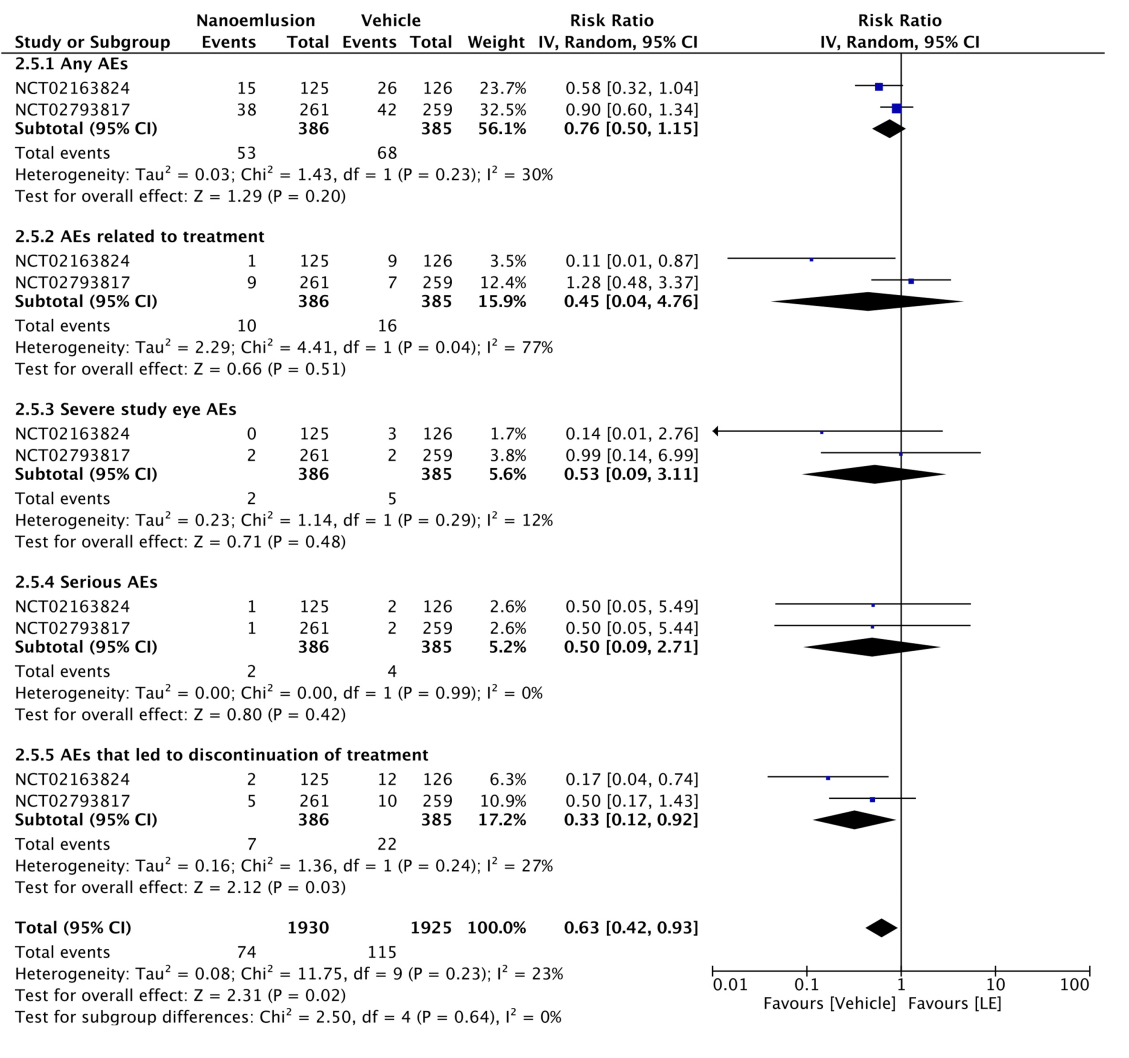
Forest plot of safety profile of 1.00% LE LE: loteprednol etabonate, CI: confidence interval; IV: inverse variance; SD: standard deviation Study or subgroup: NCT02163824 [13]; NCT02793817 [14]

Regarding safety, 0.38% LE gel was associated with minimal ocular AEs, with only one occurrence reported among 342 patients compared to two in the vehicle group (RR = 0.42, 95% CI = 0.05-3.42, P = 0.420; I² = 0%). The safety profile of 1.00% LE gel was evaluated in two RCTs. The incidences of total AEs, treatment-related AEs, SAEs, and AEs leading to treatment discontinuation were lower in the 1.00% LE group than in the vehicle group. The pooled analysis of these RCTs was significant (RR = 0.63, 95% CI = 0.42-0.93, P = 0.020; I² = 0%), indicating the 1.00% LE gel was associated with a lower risk of AEs than the vehicle (Figures 9, 10). The certainty of evidence was rated as moderate for Figure 9 and high for Figure 10, as detailed in Table 2.

Discussion 

This systematic review and meta-analysis evaluated the safety and efficacy of nanoparticle-sized 0.38% and 1.00% LE for managing pain and inflammation after cataract surgery [19]. This meta-analysis revealed significant improvements in all measured efficacy outcomes, including ACC and ACF resolution, with 0.38% and 1.00% LE showing particular promise. Notably, the safety analysis revealed a reduced risk of AEs in the intervention group, suggesting a favorable safety profile associated with using 0.38% and 1.00% LE in this context. Extensive research has provided compelling evidence supporting the effectiveness of corticosteroid eyedrops in reducing inflammation and alleviating pain after cataract surgery. However, it is imperative to acknowledge that despite their pivotal role in managing postoperative complications, ophthalmic steroid use is associated with various AEs [20,21]. Furthermore, the biochemical properties and limited bioavailability of large-particle ophthalmic steroids have been identified as impeding their dissolution and penetration, ultimately leading to suboptimal treatment outcomes [22]. In order to address this limitation, increasing studies have explored using mucoadhesive formulations and ophthalmic solutions with smaller particle sizes. These innovative formulations have shown promising results, with improved dissolution, enhanced transcorneal penetration, and significantly reduced AEs. Consequently, these findings highlight the potential benefits of using mucoadhesive formulations and ophthalmic solutions with smaller particle sizes in managing pain and inflammation after cataract surgery, ultimately resulting in improved treatment efficacy and optimized patient outcomes [23,24]. Moreover, the efficacy and safety of nanoparticle-sized LE have been extensively evaluated in the context of cataract surgery. Incorporating mucoadhesive-coated nanoparticles in LE formulations has garnered significant attention [11-15,24].

Our meta-analysis has provided valuable insights into the efficacy of a 0.38% LE gel in improving anterior chamber inflammation after cataract surgery. Despite considerable heterogeneity among the included RCTs, we observed a significant improvement in the resolution of anterior chamber inflammation with a 0.38% LE gel. Subgroup analyses revealed significantly improved anterior chamber inflammation resolution on postoperative days nine and 15. However, these findings notably showed substantial heterogeneity, implying potential variations across the included RCTs.

Our analysis also evaluated the efficacy of a 1.00% LE gel in mitigating anterior chamber inflammation after cataract surgery. Significantly reduced anterior chamber inflammation was observed, particularly on postoperative days eight and 15. Our findings on the efficacy of a lower concentration of nanoparticle-sized LE are consistent with previously published evidence supporting the effectiveness of micron-sized drug particles in 0.5% LE solutions for reducing pain and inflammation after cataract extraction [25-29]. Despite the lower LE concentration in the nanoparticle-sized formulation, our results demonstrate a comparable ability to alleviate pain and inflammation, indicating that nanoparticle-sized formulations may offer similar therapeutic benefits to conventional micron-sized formulations.

Furthermore, our study findings support the significantly better efficacy of a 0.38% LE gel in enhancing ACF resolution compared to the vehicle. Notably, the difference was statistically significant at all examined time points, underscoring the consistent and clinically meaningful impact of a 0.38% LE gel on ACF resolution. However, it is important to note that appreciable heterogeneity was observed on postoperative days 15 and 18, suggesting potential variations or divergent outcomes among the included RCTs at these time points. Additionally, the analysis of combined ACC and ACF outcomes with a 0.38% LE gel yielded significant results, albeit with significant heterogeneity. These findings are consistent with the existing literature that supports the effectiveness of micron-sized 0.5% LE formulations [25-29].

Our analysis yielded compelling results indicating that administering a 0.38% LE gel improved complete ocular pain resolution (grade = 0) after cataract surgery, emphasizing its favorable tolerability. Subgroup analyses showed significantly improved pain resolution on postoperative days three, eight, and 15. Regarding safety, our findings demonstrated that a 0.38% LE gel caused few ocular AEs among study participants. These results are consistent with previous studies examining the tolerability of 0.5% LE formulations in similar clinical contexts, which reported comparable tolerability [25-30]. Notably, our findings demonstrated significantly reduced safety concerns, with very few ocular AEs reported, compared to 0.5% LE formulations, suggesting that a 0.38% LE gel might be a safer alternative to 0.5% LE formulations regarding ocular AEs. The underlying factor contributing to this significant reduction in AEs might be the innovative formulation strategy used for nanoparticle-sized 0.38% LE, which facilitates enhanced transcorneal penetration while considerably minimizing the occurrence of AEs [24].

This study had several limitations that should be acknowledged. Firstly, relatively few RCTs were available for inclusion. This scarcity can be attributed to the novelty of the investigated intervention and the use of an innovative formulation. Therefore, the limited pool of RCTs may restrict the generalizability and robustness of our findings. Secondly, the high heterogeneity in the pooled results suggests differences in methodologies, patients, or other unreported factors among studies. However, we thoroughly explored heterogeneity sources, and the included RCTs used consistent protocols, enhancing the validity of our meta-analysis. Lastly, there is no analysis of macular edema, which is one of the primary reasons for using topical corticosteroids post-operatively.

## Conclusions

In conclusion, nanoparticle-sized 0.38% and 1.00% LE effectively reduced postoperative pain and inflammation in patients after cataract surgery. These findings support nanoparticle-sized LE as a potential therapeutic option for managing postoperative inflammation. The nanoparticle-sized LE formulation offers improved ocular tissue penetration and efficacy, potentially enhancing patient compliance and clinical outcomes. However, additional research and larger-scale studies are needed to validate these findings and explore long-term safety and efficacy.
